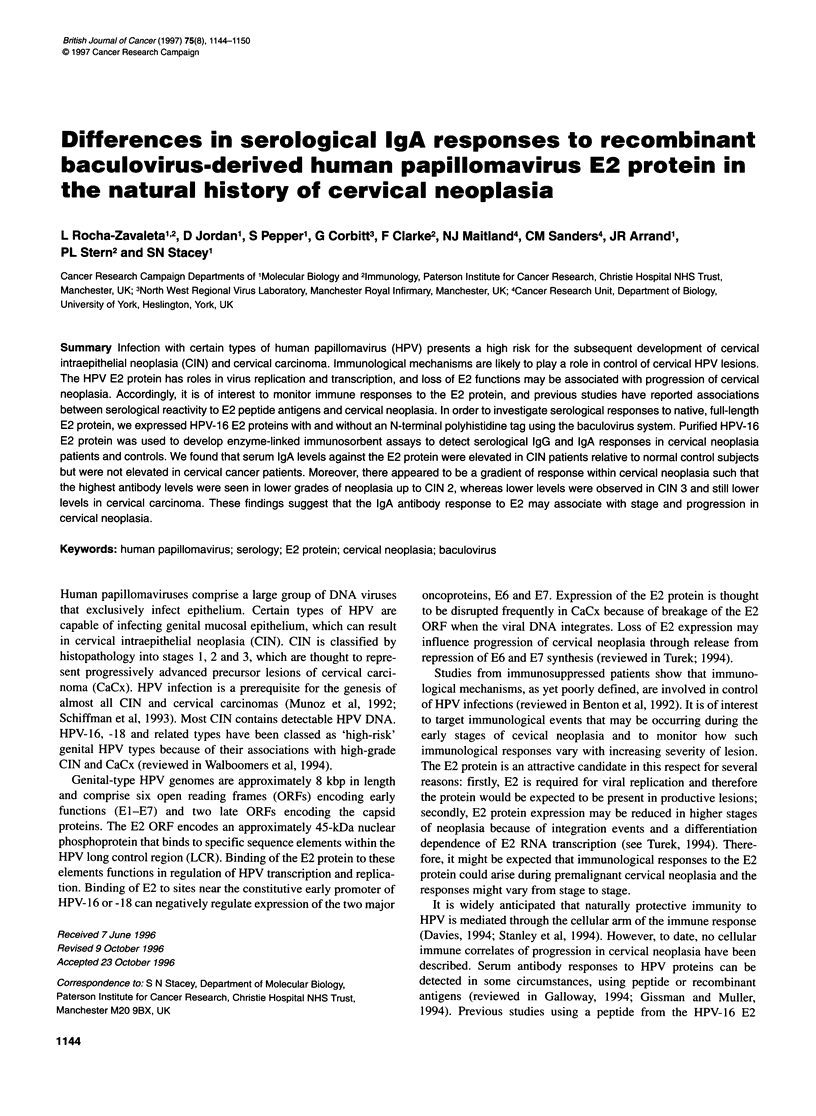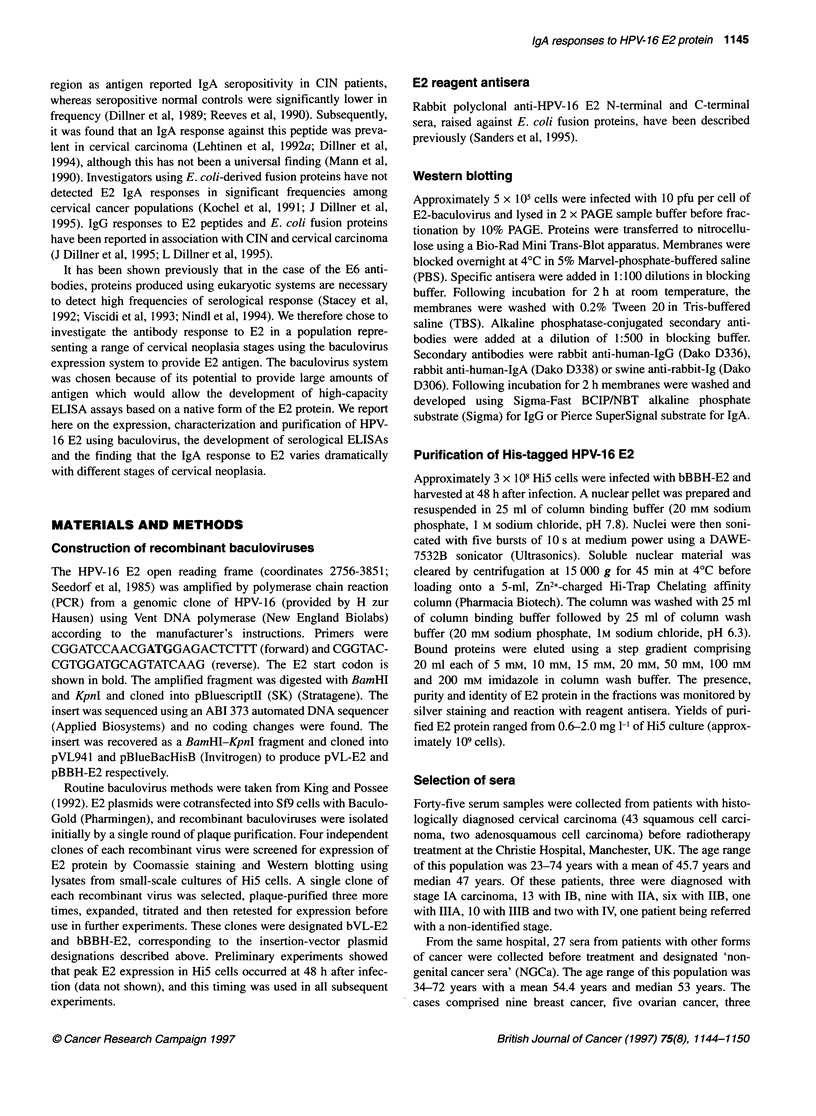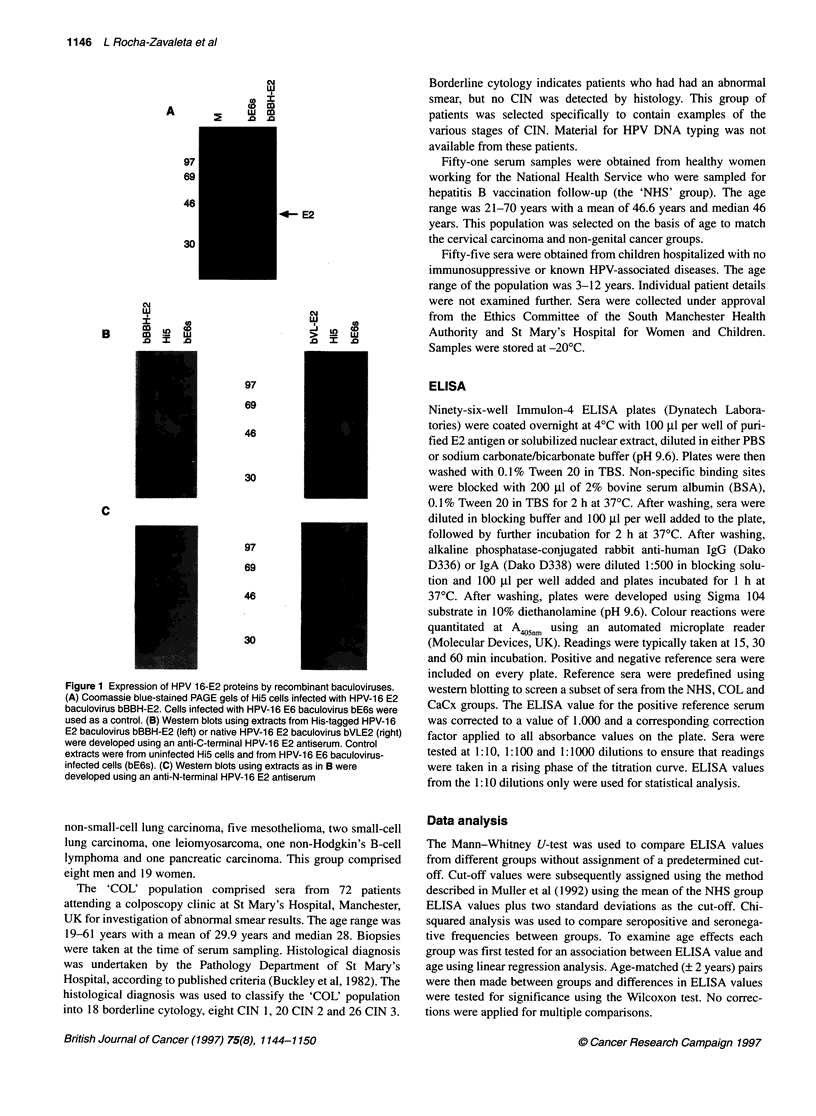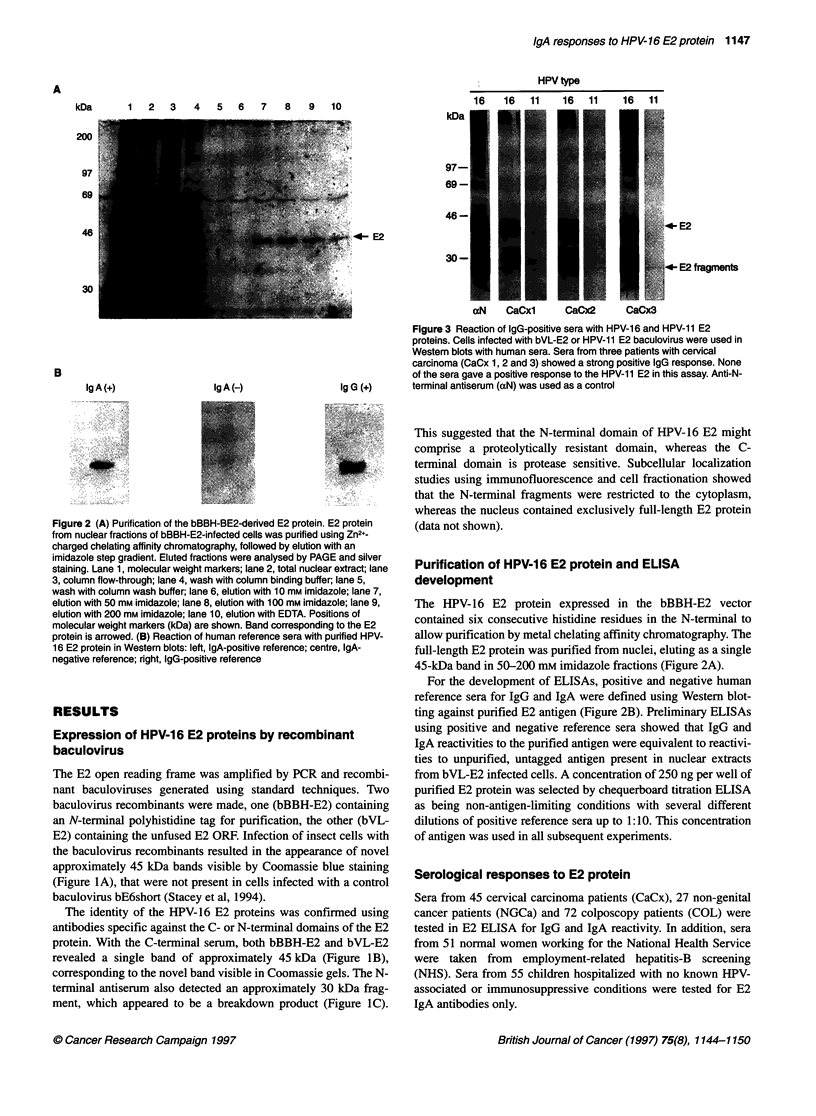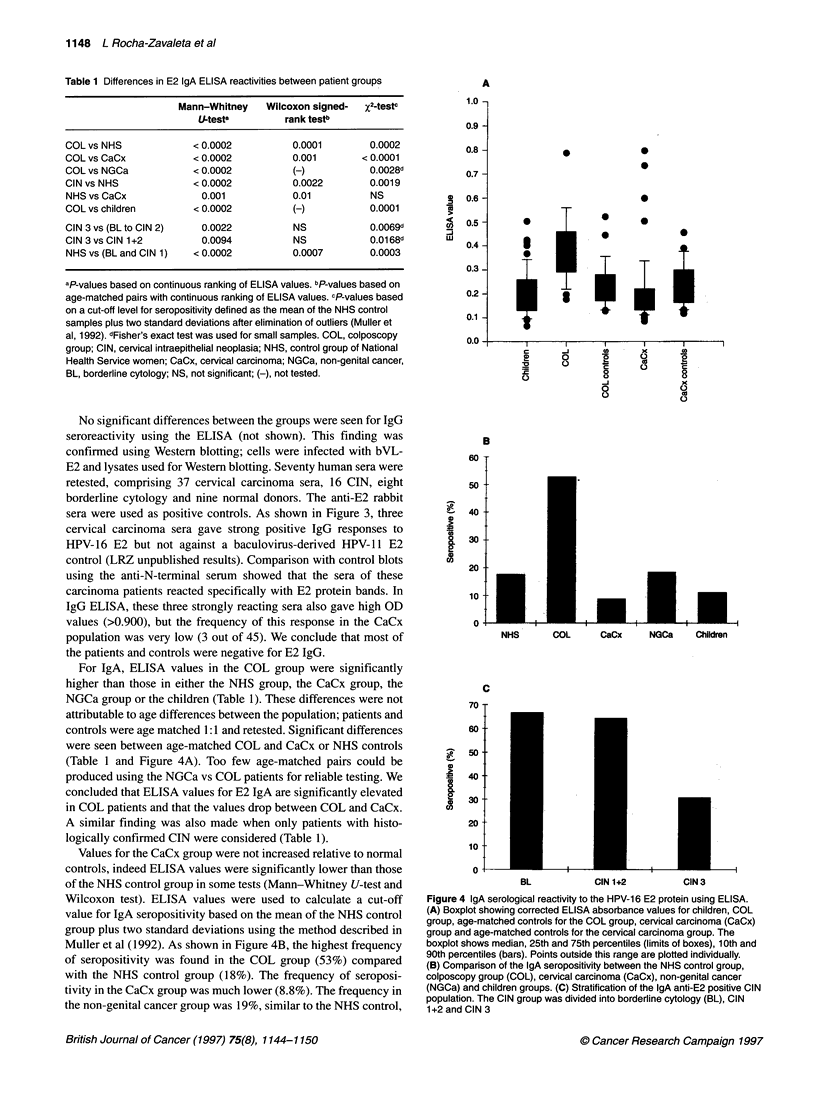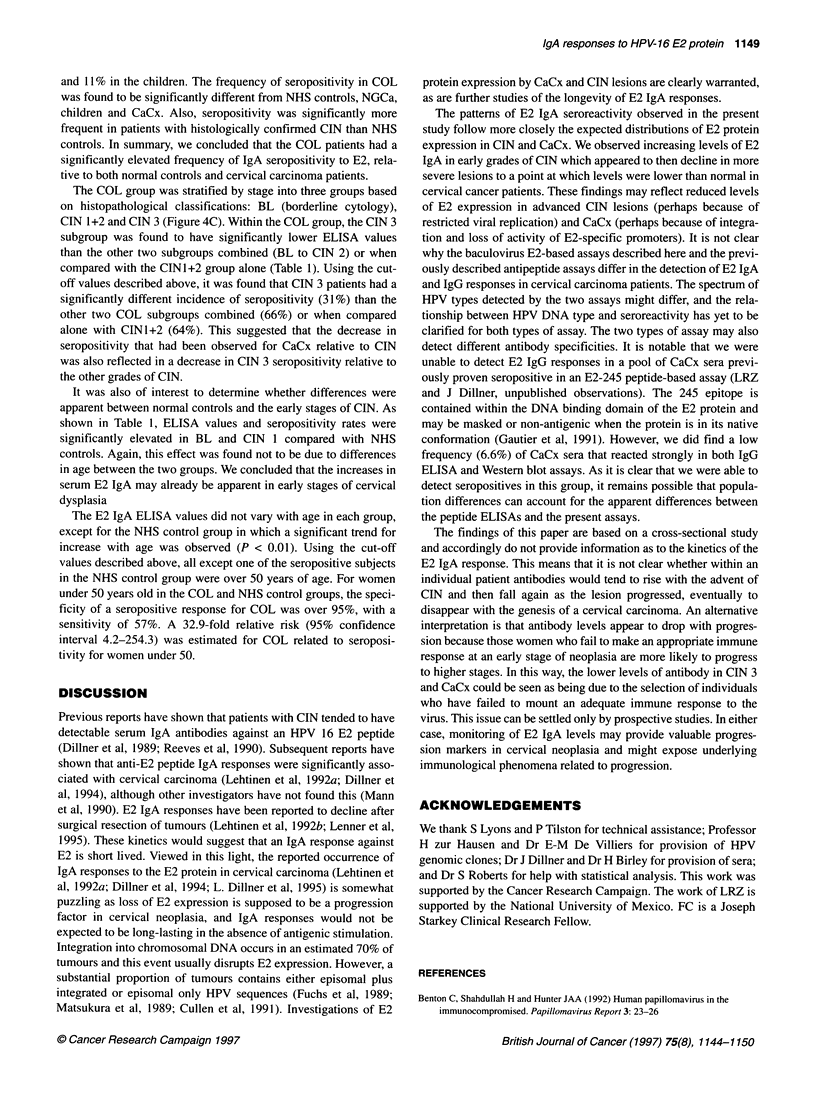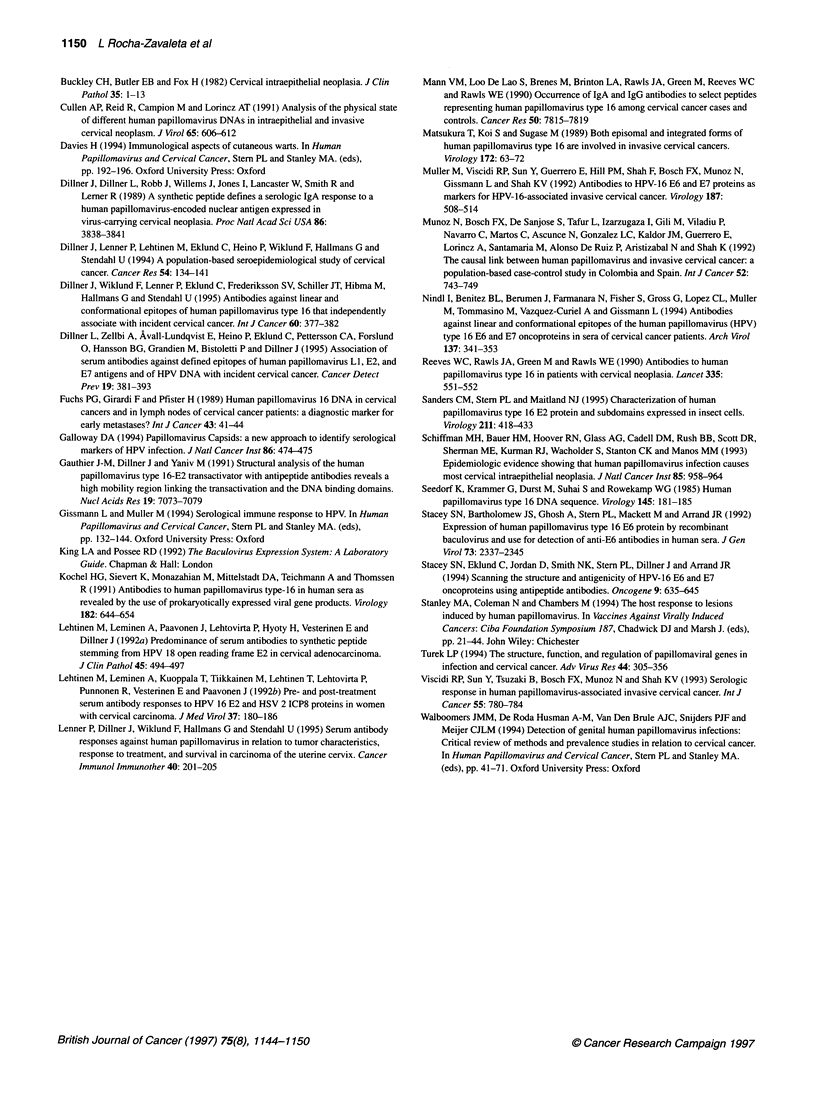# Differences in serological IgA responses to recombinant baculovirus-derived human papillomavirus E2 protein in the natural history of cervical neoplasia.

**DOI:** 10.1038/bjc.1997.197

**Published:** 1997

**Authors:** L. Rocha-Zavaleta, D. Jordan, S. Pepper, G. Corbitt, F. Clarke, N. J. Maitland, C. M. Sanders, J. R. Arrand, P. L. Stern, S. N. Stacey

**Affiliations:** Department of Molecular Biology, Paterson Institute for Cancer Research, Christie Hospital NHS Trust, Manchester, UK.

## Abstract

**Images:**


British Journal of Cancer (1997) 75(8), 1144–1150
© 1997 Cancer Research Campaign

### Differences in serological IgA responses to recombinant baculovirus-derived human papillomavirus E2 protein in the natural history of cervical neoplasia

**L Rocha-Zavaleta[1,2], D Jordan[1], S Pepper[1], G Corbitt[3], F Clarke[2], NJ Maitland[4], CM Sanders[4], JR Arrand[1], PL Stern[2] and SN Stacey[1]**

Cancer Research Campaign Departments of [1]Molecular Biology and [2]Immunology, Paterson Institute for Cancer Research, Christie Hospital NHS Trust, Manchester, UK; [3]North West Regional Virus Laboratory, Manchester Royal Infirmary, Manchester, UK; [4]Cancer Research Unit, Department of Biology, University of York, Heslington, York, UK

**Summary** Infection with certain types of human papillomavirus (HPV) presents a high risk for the subsequent development of cervical intraepithelial neoplasia (CIN) and cervical carcinoma. Immunological mechanisms are likely to play a role in control of cervical HPV lesions. The HPV E2 protein has roles in virus replication and transcription, and loss of E2 functions may be associated with progression of cervical neoplasia. Accordingly, it is of interest to monitor immune responses to the E2 protein, and previous studies have reported associations between serological reactivity to E2 peptide antigens and cervical neoplasia. In order to investigate serological responses to native, full-length E2 protein, we expressed HPV-16 E2 proteins with and without an N-terminal polyhistidine tag using the baculovirus system. Purified HPV-16 E2 protein was used to develop enzyme-linked immunosorbent assays to detect serological IgG and IgA responses in cervical neoplasia patients and controls. We found that serum IgA levels against the E2 protein were elevated in CIN patients relative to normal control subjects but were not elevated in cervical cancer patients. Moreover, there appeared to be a gradient of response within cervical neoplasia such that the highest antibody levels were seen in lower grades of neoplasia up to CIN 2, whereas lower levels were observed in CIN 3 and still lower levels in cervical carcinoma. These findings suggest that the IgA antibody response to E2 may associate with stage and progression in cervical neoplasia.

**Keywords:** human papillomavirus; serology; E2 protein; cervical neoplasia; baculovirus

Human papillomaviruses comprise a large group of DNA viruses that exclusively infect epithelium. Certain types of HPV are capable of infecting genital mucosal epithelium, which can result in cervical intraepithelial neoplasia (CIN). CIN is classified by histopathology into stages 1, 2 and 3, which are thought to represent progressively advanced precursor lesions of cervical carcinoma (CaCx). HPV infection is a prerequisite for the genesis of almost all CIN and cervical carcinomas (Munoz et al, 1992; Schiffman et al, 1993). Most CIN contains detectable HPV DNA. HPV-16, -18 and related types have been classed as 'high-risk' genital HPV types because of their associations with high-grade CIN and CaCx (reviewed in Walboomers et al, 1994).

Genital-type HPV genomes are approximately 8 kbp in length and comprise six open reading frames (ORFs) encoding early functions (E1–E7) and two late ORFs encoding the capsid proteins. The E2 ORF encodes an approximately 45-kDa nuclear phosphoprotein that binds to specific sequence elements within the HPV long control region (LCR). Binding of the E2 protein to these elements functions in regulation of HPV transcription and replication. Binding of E2 to sites near the constitutive early promoter of HPV-16 or -18 can negatively regulate expression of the two major oncoproteins, E6 and E7. Expression of the E2 protein is thought to be disrupted frequently in CaCx because of breakage of the E2 ORF when the viral DNA integrates. Loss of E2 expression may influence progression of cervical neoplasia through release from repression of E6 and E7 synthesis (reviewed in Turek; 1994).

Studies from immunosuppressed patients show that immunological mechanisms, as yet poorly defined, are involved in control of HPV infections (reviewed in Benton et al, 1992). It is of interest to target immunological events that may be occurring during the early stages of cevical neoplasia and to monitor how such immunological responses vary with increasing severity of lesion. The E2 protein is an attractive candidate in this respect for several reasons: firstly, E2 is required for viral replication and therefore the protein would be expected to be present in productive lesions; secondly, E2 protein expression may be reduced in higher stages of neoplasia because of integration events and a differentiation dependence of E2 RNA transcription (see Turek, 1994). Therefore, it might be expected that immunological responses to the E2 protein could arise during premalignant cervical neoplasia and the responses might vary from stage to stage.

It is widely anticipated that naturally protective immunity to HPV is mediated through the cellular arm of the immune response (Davies, 1994; Stanley et al, 1994). However, to date, no cellular immune correlates of progression in cervical neoplasia have been described. Serum antibody responses to HPV proteins can be detected in some circumstances, using peptide or recombinant antigens (reviewed in Galloway, 1994; Gissman and Muller, 1994). Previous studies using a peptide from the HPV-16 E2

*Received 7 June 1996*
*Revised 9 October 1996*
*Accepted 23 October 1996*

*Correspondence to:* S N Stacey, Department of Molecular Biology, Paterson Institute for Cancer Research, Christie Hospital NHS Trust, Manchester M20 9BX, UK

region as antigen reported IgA seropositivity in CIN patients, whereas seropositive normal controls were significantly lower in frequency (Dillner et al, 1989; Reeves et al, 1990). Subsequently, it was found that an IgA response against this peptide was prevalent in cervical carcinoma (Lehtinen et al, 1992*a*; Dillner et al, 1994), although this has not been a universal finding (Mann et al, 1990). Investigators using *E. coli*-derived fusion proteins have not detected E2 IgA responses in significant frequencies among cervical cancer populations (Kochel et al, 1991; J Dillner et al, 1995). IgG responses to E2 peptides and *E. coli* fusion proteins have been reported in association with CIN and cervical carcinoma (J Dillner et al, 1995; L Dillner et al, 1995).

It has been shown previously that in the case of the E6 antibodies, proteins produced using eukaryotic systems are necessary to detect high frequencies of serological response (Stacey et al, 1992; Viscidi et al, 1993; Nindl et al, 1994). We therefore chose to investigate the antibody response to E2 in a population representing a range of cervical neoplasia stages using the baculovirus expression system to provide E2 antigen. The baculovirus system was chosen because of its potential to provide large amounts of antigen which would allow the development of high-capacity ELISA assays based on a native form of the E2 protein. We report here on the expression, characterization and purification of HPV-16 E2 using baculovirus, the development of serological ELISAs and the finding that the IgA response to E2 varies dramatically with different stages of cervical neoplasia.

### MATERIALS AND METHODS

### Construction of recombinant baculoviruses

The HPV-16 E2 open reading frame (coordinates 2756-3851; Seedorf et al, 1985) was amplified by polymerase chain reaction (PCR) from a genomic clone of HPV-16 (provided by H zur Hausen) using Vent DNA polymerase (New England Biolabs) according to the manufacturer's instructions. Primers were CGGATCCAACG**ATG**GAGACTCTTT (forward) and CGGTACCGTGGATGCAGTATCAAG (reverse). The E2 start codon is shown in bold. The amplified fragment was digested with *Bam*HI and *Kpn*I and cloned into pBluescriptII (SK) (Stratagene). The insert was sequenced using an ABI 373 automated DNA sequencer (Applied Biosystems) and no coding changes were found. The insert was recovered as a *Bam*HI–*Kpn*I fragment and cloned into pVL941 and pBlueBacHisB (Invitrogen) to produce pVL-E2 and pBBH-E2 respectively.

Routine baculovirus methods were taken from King and Possee (1992). E2 plasmids were cotransfected into Sf9 cells with BaculoGold (Pharmingen), and recombinant baculoviruses were isolated initially by a single round of plaque purification. Four independent clones of each recombinant virus were screened for expression of E2 protein by Coomassie staining and Western blotting using lysates from small-scale cultures of Hi5 cells. A single clone of each recombinant virus was selected, plaque-purified three more times, expanded, titrated and then retested for expression before use in further experiments. These clones were designated bVL-E2 and bBBH-E2, corresponding to the insertion-vector plasmid designations described above. Preliminary experiments showed that peak E2 expression in Hi5 cells occurred at 48 h after infection (data not shown), and this timing was used in all subsequent experiments.

### E2 reagent antisera

Rabbit polyclonal anti-HPV-16 E2 N-terminal and C-terminal sera, raised against *E. coli* fusion proteins, have been described previously (Sanders et al, 1995).

### Western blotting

Approximately $5 \times 10^5$ cells were infected with 10 pfu per cell of E2-baculovirus and lysed in 2 × PAGE sample buffer before fractionation by 10% PAGE. Proteins were transferred to nitrocellulose using a Bio-Rad Mini Trans-Blot apparatus. Membranes were blocked overnight at 4°C in 5% Marvel-phosphate-buffered saline (PBS). Specific antisera were added in 1:100 dilutions in blocking buffer. Following incubation for 2 h at room temperature, the membranes were washed with 0.2% Tween 20 in Tris-buffered saline (TBS). Alkaline phosphatase-conjugated secondary antibodies were added at a dilution of 1:500 in blocking buffer. Secondary antibodies were rabbit anti-human-IgG (Dako D336), rabbit anti-human-IgA (Dako D338) or swine anti-rabbit-Ig (Dako D306). Following incubation for 2 h membranes were washed and developed using Sigma-Fast BCIP/NBT alkaline phosphate substrate (Sigma) for IgG or Pierce SuperSignal substrate for IgA.

### Purification of His-tagged HPV-16 E2

Approximately $3 \times 10^8$ Hi5 cells were infected with bBBH-E2 and harvested at 48 h after infection. A nuclear pellet was prepared and resuspended in 25 ml of column binding buffer (20 mM sodium phosphate, 1 M sodium chloride, pH 7.8). Nuclei were then sonicated with five bursts of 10 s at medium power using a DAWE-7532B sonicator (Ultrasonics). Soluble nuclear material was cleared by centrifugation at 15 000 *g* for 45 min at 4°C before loading onto a 5-ml, $Zn^{2+}$-charged Hi-Trap Chelating affinity column (Pharmacia Biotech). The column was washed with 25 ml of column binding buffer followed by 25 ml of column wash buffer (20 mM sodium phosphate, 1M sodium chloride, pH 6.3). Bound proteins were eluted using a step gradient comprising 20 ml each of 5 mM, 10 mM, 15 mM, 20 mM, 50 mM, 100 mM and 200 mM imidazole in column wash buffer. The presence, purity and identity of E2 protein in the fractions was monitored by silver staining and reaction with reagent antisera. Yields of purified E2 protein ranged from 0.6–2.0 mg $l^{-1}$ of Hi5 culture (approximately $10^9$ cells).

### Selection of sera

Forty-five serum samples were collected from patients with histologically diagnosed cervical carcinoma (43 squamous cell carcinoma, two adenosquamous cell carcinoma) before radiotherapy treatment at the Christie Hospital, Manchester, UK. The age range of this population was 23–74 years with a mean of 45.7 years and median 47 years. Of these patients, three were diagnosed with stage IA carcinoma, 13 with IB, nine with IIA, six with IIB, one with IIIA, 10 with IIIB and two with IV, one patient being referred with a non-identified stage.

From the same hospital, 27 sera from patients with other forms of cancer were collected before treatment and designated 'non-genital cancer sera' (NGCa). The age range of this population was 34–72 years with a mean 54.4 years and median 53 years. The cases comprised nine breast cancer, five ovarian cancer, three

© Cancer Research Campaign 1997

**Figure 1** Expression of HPV 16-E2 proteins by recombinant baculoviruses. (**A**) Coomassie blue-stained PAGE gels of Hi5 cells infected with HPV-16 E2 baculovirus bBBH-E2. Cells infected with HPV-16 E6 baculovirus bE6s were used as a control. (**B**) Western blots using extracts from His-tagged HPV-16 E2 baculovirus bBBH-E2 (left) or native HPV-16 E2 baculovirus bVLE2 (right) were developed using an anti-C-terminal HPV-16 E2 antiserum. Control extracts were from uninfected Hi5 cells and from HPV-16 E6 baculovirus-infected cells (bE6s). (**C**) Western blots using extracts as in **B** were developed using an anti-N-terminal HPV-16 E2 antiserum non-small-cell lung carcinoma, five mesothelioma, two small-cell lung carcinoma, one leiomyosarcoma, one non-Hodgkin's B-cell lymphoma and one pancreatic carcinoma. This group comprised eight men and 19 women.

The 'COL' population comprised sera from 72 patients attending a colposcopy clinic at St Mary's Hospital, Manchester, UK for investigation of abnormal smear results. The age range was 19–61 years with a mean of 29.9 years and median 28. Biopsies were taken at the time of serum sampling. Histological diagnosis was undertaken by the Pathology Department of St Mary's Hospital, according to published criteria (Buckley et al, 1982). The histological diagnosis was used to classify the 'COL' population into 18 borderline cytology, eight CIN 1, 20 CIN 2 and 26 CIN 3. Borderline cytology indicates patients who had had an abnormal smear, but no CIN was detected by histology. This group of patients was selected specifically to contain examples of the various stages of CIN. Material for HPV DNA typing was not available from these patients.

Fifty-one serum samples were obtained from healthy women working for the National Health Service who were sampled for hepatitis B vaccination follow-up (the 'NHS' group). The age range was 21–70 years with a mean of 46.6 years and median 46 years. This population was selected on the basis of age to match the cervical carcinoma and non-genital cancer groups.

Fifty-five sera were obtained from children hospitalized with no immunosuppressive or known HPV-associated diseases. The age range of the population was 3–12 years. Individual patient details were not examined further. Sera were collected under approval from the Ethics Committee of the South Manchester Health Authority and St Mary's Hospital for Women and Children. Samples were stored at –20°C.

### ELISA

Ninety-six-well Immulon-4 ELISA plates (Dynatech Laboratories) were coated overnight at 4°C with 100 μl per well of purified E2 antigen or solubilized nuclear extract, diluted in either PBS or sodium carbonate/bicarbonate buffer (pH 9.6). Plates were then washed with 0.1% Tween 20 in TBS. Non-specific binding sites were blocked with 200 μl of 2% bovine serum albumin (BSA), 0.1% Tween 20 in TBS for 2 h at 37°C. After washing, sera were diluted in blocking buffer and 100 μl per well added to the plate, followed by further incubation for 2 h at 37°C. After washing, alkaline phosphatase-conjugated rabbit anti-human IgG (Dako D336) or IgA (Dako D338) were diluted 1:500 in blocking solution and 100 μl per well added and plates incubated for 1 h at 37°C. After washing, plates were developed using Sigma 104 substrate in 10% diethanolamine (pH 9.6). Colour reactions were quantitated at $A_{405nm}$ using an automated microplate reader (Molecular Devices, UK). Readings were typically taken at 15, 30 and 60 min incubation. Positive and negative reference sera were included on every plate. Reference sera were predefined using western blotting to screen a subset of sera from the NHS, COL and CaCx groups. The ELISA value for the positive reference serum was corrected to a value of 1.000 and a corresponding correction factor applied to all absorbance values on the plate. Sera were tested at 1:10, 1:100 and 1:1000 dilutions to ensure that readings were taken in a rising phase of the titration curve. ELISA values from the 1:10 dilutions only were used for statistical analysis.

### Data analysis

The Mann–Whitney *U*-test was used to compare ELISA values from different groups without assignment of a predetermined cut-off. Cut-off values were subsequently assigned using the method described in Muller et al (1992) using the mean of the NHS group ELISA values plus two standard deviations as the cut-off. Chi-squared analysis was used to compare seropositive and seronegative frequencies between groups. To examine age effects each group was first tested for an association between ELISA value and age using linear regression analysis. Age-matched (± 2 years) pairs were then made between groups and differences in ELISA values were tested for significance using the Wilcoxon test. No corrections were applied for multiple comparisons.

*© Cancer Research Campaign 1997*

**Figure 2** (**A**) Purification of the bBBH-BE2-derived E2 protein. E2 protein from nuclear fractions of bBBH-E2-infected cells was purified using $Zn^{2+}$-charged chelating affinity chromatography, followed by elution with an imidazole step gradient. Eluted fractions were analysed by PAGE and silver staining. Lane 1, molecular weight markers; lane 2, total nuclear extract; lane 3, column flow-through; lane 4, wash with column binding buffer; lane 5, wash with column wash buffer; lane 6, elution with 10 mM imidazole; lane 7, elution with 50 mM imidazole; lane 8, elution with 100 mM imidazole; lane 9, elution with 200 mM imidazole; lane 10, elution with EDTA. Positions of molecular weight markers (kDa) are shown. Band corresponding to the E2 protein is arrowed. (**B**) Reaction of human reference sera with purified HPV-16 E2 protein in Western blots: left, IgA-positive reference; centre, IgA-negative reference; right, IgG-positive reference

### RESULTS

### Expression of HPV-16 E2 proteins by recombinant baculovirus

The E2 open reading frame was amplified by PCR and recombinant baculoviruses generated using standard techniques. Two baculovirus recombinants were made, one (bBBH-E2) containing an *N*-terminal polyhistidine tag for purification, the other (bVL-E2) containing the unfused E2 ORF. Infection of insect cells with the baculovirus recombinants resulted in the appearance of novel approximately 45 kDa bands visible by Coomassie blue staining (Figure 1A), that were not present in cells infected with a control baculovirus bE6short (Stacey et al, 1994).

The identity of the HPV-16 E2 proteins was confirmed using antibodies specific against the C- or N-terminal domains of the E2 protein. With the C-terminal serum, both bBBH-E2 and bVL-E2 revealed a single band of approximately 45 kDa (Figure 1B), corresponding to the novel band visible in Coomassie gels. The N-terminal antiserum also detected an approximately 30 kDa fragment, which appeared to be a breakdown product (Figure 1C).

**Figure 3** Reaction of IgG-positive sera with HPV-16 and HPV-11 E2 proteins. Cells infected with bVL-E2 or HPV-11 E2 baculovirus were used in Western blots with human sera. Sera from three patients with cervical carcinoma (CaCx 1, 2 and 3) showed a strong positive IgG response. None of the sera gave a positive response to the HPV-11 E2 in this assay. Anti-N-terminal antiserum (αN) was used as a control

This suggested that the N-terminal domain of HPV-16 E2 might comprise a proteolytically resistant domain, whereas the C-terminal domain is protease sensitive. Subcellular localization studies using immunofluorescence and cell fractionation showed that the N-terminal fragments were restricted to the cytoplasm, whereas the nucleus contained exclusively full-length E2 protein (data not shown).

### Purification of HPV-16 E2 protein and ELISA development

The HPV-16 E2 protein expressed in the bBBH-E2 vector contained six consecutive histidine residues in the N-terminal to allow purification by metal chelating affinity chromatography. The full-length E2 protein was purified from nuclei, eluting as a single 45-kDa band in 50–200 mM imidazole fractions (Figure 2A).

For the development of ELISAs, positive and negative human reference sera for IgG and IgA were defined using Western blotting against purified E2 antigen (Figure 2B). Preliminary ELISAs using positive and negative reference sera showed that IgG and IgA reactivities to the purified antigen were equivalent to reactivities to unpurified, untagged antigen present in nuclear extracts from bVL-E2 infected cells. A concentration of 250 ng per well of purified E2 protein was selected by chequerboard titration ELISA as being non-antigen-limiting conditions with several different dilutions of positive reference sera up to 1:10. This concentration of antigen was used in all subsequent experiments.

### Serological responses to E2 protein

Sera from 45 cervical carcinoma patients (CaCx), 27 non-genital cancer patients (NGCa) and 72 colposcopy patients (COL) were tested in E2 ELISA for IgG and IgA reactivity. In addition, sera from 51 normal women working for the National Health Service were taken from employment-related hepatitis-B screening (NHS). Sera from 55 children hospitalized with no known HPV-associated or immunosuppressive conditions were tested for E2 IgA antibodies only.

**Table 1** Differences in E2 IgA ELISA reactivities between patient groups

| | Mann–Whitney *U*-test[a] | Wilcoxon signed-rank test[b] | $\chi^2$-test[c] |
|---|---|---|---|
| COL vs NHS | < 0.0002 | 0.0001 | 0.0002 |
| COL vs CaCx | < 0.0002 | 0.001 | < 0.0001 |
| COL vs NGCa | < 0.0002 | (–) | 0.0028[d] |
| CIN vs NHS | < 0.0002 | 0.0022 | 0.0019 |
| NHS vs CaCx | 0.001 | 0.01 | NS |
| COL vs children | < 0.0002 | (–) | 0.0001 |
| CIN 3 vs (BL to CIN 2) | 0.0022 | NS | 0.0069[d] |
| CIN 3 vs CIN 1+2 | 0.0094 | NS | 0.0168[d] |
| NHS vs (BL and CIN 1) | < 0.0002 | 0.0007 | 0.0003 |

[a]*P*-values based on continuous ranking of ELISA values. [b]*P*-values based on age-matched pairs with continuous ranking of ELISA values. [c]*P*-values based on a cut-off level for seropositivity defined as the mean of the NHS control samples plus two standard deviations after elimination of outliers (Muller et al, 1992). [d]Fisher's exact test was used for small samples. COL, colposcopy group; CIN, cervical intraepithelial neoplasia; NHS, control group of National Health Service women; CaCx, cervical carcinoma; NGCa, non-genital cancer, BL, borderline cytology; NS, not significant; (–), not tested.

No significant differences between the groups were seen for IgG seroreactivity using the ELISA (not shown). This finding was confirmed using Western blotting; cells were infected with bVL-E2 and lysates used for Western blotting. Seventy human sera were retested, comprising 37 cervical carcinoma sera, 16 CIN, eight borderline cytology and nine normal donors. The anti-E2 rabbit sera were used as positive controls. As shown in Figure 3, three cervical carcinoma sera gave strong positive IgG responses to HPV-16 E2 but not against a baculovirus-derived HPV-11 E2 control (LRZ unpublished results). Comparison with control blots using the anti-N-terminal serum showed that the sera of these carcinoma patients reacted specifically with E2 protein bands. In IgG ELISA, these three strongly reacting sera also gave high OD values (>0.900), but the frequency of this response in the CaCx population was very low (3 out of 45). We conclude that most of the patients and controls were negative for E2 IgG.

For IgA, ELISA values in the COL group were significantly higher than those in either the NHS group, the CaCx group, the NGCa group or the children (Table 1). These differences were not attributable to age differences between the population; patients and controls were age matched 1:1 and retested. Significant differences were seen between age-matched COL and CaCx or NHS controls (Table 1 and Figure 4A). Too few age-matched pairs could be produced using the NGCa vs COL patients for reliable testing. We concluded that ELISA values for E2 IgA are significantly elevated in COL patients and that the values drop between COL and CaCx. A similar finding was also made when only patients with histologically confirmed CIN were considered (Table 1).

Values for the CaCx group were not increased relative to normal controls, indeed ELISA values were significantly lower than those of the NHS control group in some tests (Mann–Whitney *U*-test and Wilcoxon test). ELISA values were used to calculate a cut-off value for IgA seropositivity based on the mean of the NHS control group plus two standard deviations using the method described in Muller et al (1992). As shown in Figure 4B, the highest frequency of seropositivity was found in the COL group (53%) compared with the NHS control group (18%). The frequency of seropositivity in the CaCx group was much lower (8.8%). The frequency in the non-genital cancer group was 19%, similar to the NHS control,

**Figure 4** IgA serological reactivity to the HPV-16 E2 protein using ELISA. **(A)** Boxplot showing corrected ELISA absorbance values for children, COL group, age-matched controls for the COL group, cervical carcinoma (CaCx) group and age-matched controls for the cervical carcinoma group. The boxplot shows median, 25th and 75th percentiles (limits of boxes), 10th and 90th percentiles (bars). Points outside this range are plotted individually. **(B)** Comparison of the IgA seropositivity between the NHS control group, colposcopy group (COL), cervical carcinoma (CaCx), non-genital cancer (NGCa) and children groups. **(C)** Stratification of the IgA anti-E2 positive CIN population. The CIN group was divided into borderline cytology (BL), CIN 1+2 and CIN 3

© Cancer Research Campaign 1997

and 11% in the children. The frequency of seropositivity in COL was found to be significantly different from NHS controls, NGCa, children and CaCx. Also, seropositivity was significantly more frequent in patients with histologically confirmed CIN than NHS controls. In summary, we concluded that the COL patients had a significantly elevated frequency of IgA seropositivity to E2, relative to both normal controls and cervical carcinoma patients.

The COL group was stratified by stage into three groups based on histopathological classifications: BL (borderline cytology), CIN 1+2 and CIN 3 (Figure 4C). Within the COL group, the CIN 3 subgroup was found to have significantly lower ELISA values than the other two subgroups combined (BL to CIN 2) or when compared with the CIN1+2 group alone (Table 1). Using the cut-off values described above, it was found that CIN 3 patients had a significantly different incidence of seropositivity (31%) than the other two COL subgroups combined (66%) or when compared alone with CIN1+2 (64%). This suggested that the decrease in seropositivity that had been observed for CaCx relative to CIN was also reflected in a decrease in CIN 3 seropositivity relative to the other grades of CIN.

It was also of interest to determine whether differences were apparent between normal controls and the early stages of CIN. As shown in Table 1, ELISA values and seropositivity rates were significantly elevated in BL and CIN 1 compared with NHS controls. Again, this effect was found not to be due to differences in age between the two groups. We concluded that the increases in serum E2 IgA may already be apparent in early stages of cervical dysplasia

The E2 IgA ELISA values did not vary with age in each group, except for the NHS control group in which a significant trend for increase with age was observed ($P < 0.01$). Using the cut-off values described above, all except one of the seropositive subjects in the NHS control group were over 50 years of age. For women under 50 years old in the COL and NHS control groups, the specificity of a seropositive response for COL was over 95%, with a sensitivity of 57%. A 32.9-fold relative risk (95% confidence interval 4.2–254.3) was estimated for COL related to seropositivity for women under 50.

### DISCUSSION

Previous reports have shown that patients with CIN tended to have detectable serum IgA antibodies against an HPV 16 E2 peptide (Dillner et al, 1989; Reeves et al, 1990). Subsequent reports have shown that anti-E2 peptide IgA responses were significantly associated with cervical carcinoma (Lehtinen et al, 1992*a*; Dillner et al, 1994), although other investigators have not found this (Mann et al, 1990). E2 IgA responses have been reported to decline after surgical resection of tumours (Lehtinen et al, 1992*b*; Lenner et al, 1995). These kinetics would suggest that an IgA response against E2 is short lived. Viewed in this light, the reported occurrence of IgA responses to the E2 protein in cervical carcinoma (Lehtinen et al, 1992*a*; Dillner et al, 1994; L. Dillner et al, 1995) is somewhat puzzling as loss of E2 expression is supposed to be a progression factor in cervical neoplasia, and IgA responses would not be expected to be long-lasting in the absence of antigenic stimulation. Integration into chromosomal DNA occurs in an estimated 70% of tumours and this event usually disrupts E2 expression. However, a substantial proportion of tumours contains either episomal plus integrated or episomal only HPV sequences (Fuchs et al, 1989; Matsukura et al, 1989; Cullen et al, 1991). Investigations of E2 protein expression by CaCx and CIN lesions are clearly warranted, as are further studies of the longevity of E2 IgA responses.

The patterns of E2 IgA seroreactivity observed in the present study follow more closely the expected distributions of E2 protein expression in CIN and CaCx. We observed increasing levels of E2 IgA in early grades of CIN which appeared to then decline in more severe lesions to a point at which levels were lower than normal in cervical cancer patients. These findings may reflect reduced levels of E2 expression in advanced CIN lesions (perhaps because of restricted viral replication) and CaCx (perhaps because of integration and loss of activity of E2-specific promoters). It is not clear why the baculovirus E2-based assays described here and the previously described antipeptide assays differ in the detection of E2 IgA and IgG responses in cervical carcinoma patients. The spectrum of HPV types detected by the two assays might differ, and the relationship between HPV DNA type and seroreactivity has yet to be clarified for both types of assay. The two types of assay may also detect different antibody specificities. It is notable that we were unable to detect E2 IgG responses in a pool of CaCx sera previously proven seropositive in an E2-245 peptide-based assay (LRZ and J Dillner, unpublished observations). The 245 epitope is contained within the DNA binding domain of the E2 protein and may be masked or non-antigenic when the protein is in its native conformation (Gautier et al, 1991). However, we did find a low frequency (6.6%) of CaCx sera that reacted strongly in both IgG ELISA and Western blot assays. As it is clear that we were able to detect seropositives in this group, it remains possible that population differences can account for the apparent differences between the peptide ELISAs and the present assays.

The findings of this paper are based on a cross-sectional study and accordingly do not provide information as to the kinetics of the E2 IgA response. This means that it is not clear whether within an individual patient antibodies would tend to rise with the advent of CIN and then fall again as the lesion progressed, eventually to disappear with the genesis of a cervical carcinoma. An alternative interpretation is that antibody levels appear to drop with progression because those women who fail to make an appropriate immune response at an early stage of neoplasia are more likely to progress to higher stages. In this way, the lower levels of antibody in CIN 3 and CaCx could be seen as being due to the selection of individuals who have failed to mount an adequate immune response to the virus. This issue can be settled only by prospective studies. In either case, monitoring of E2 IgA levels may provide valuable progression markers in cervical neoplasia and might expose underlying immunological phenomena related to progression.

### ACKNOWLEDGEMENTS

We thank S Lyons and P Tilston for technical assistance; Professor H zur Hausen and Dr E-M De Villiers for provision of HPV genomic clones; Dr J Dillner and Dr H Birley for provision of sera; and Dr S Roberts for help with statistical analysis. This work was supported by the Cancer Research Campaign. The work of LRZ is supported by the National University of Mexico. FC is a Joseph Starkey Clinical Research Fellow.

### REFERENCES

Benton C, Shahdullah H and Hunter JAA (1992) Human papillomavirus in the immunocompromised. *Papillomavirus Report* 3: 23–26

© Cancer Research Campaign 1997

Buckley CH, Butler EB and Fox H (1982) Cervical intraepithelial neoplasia. *J Clin Pathol* **35**: 1–13

Cullen AP, Reid R, Campion M and Lorincz AT (1991) Analysis of the physical state of different human papillomavirus DNAs in intraepithelial and invasive cervical neoplasm. *J Virol* **65**: 606–612

Davies H (1994) Immunological aspects of cutaneous warts. In *Human Papillomavirus and Cervical Cancer*, Stern PL and Stanley MA. (eds), pp. 192–196. Oxford University Press: Oxford

Dillner J, Dillner L, Robb J, Willems J, Jones I, Lancaster W, Smith R and Lerner R (1989) A synthetic peptide defines a serologic IgA response to a human papillomavirus-encoded nuclear antigen expressed in virus-carrying cervical neoplasia. *Proc Natl Acad Sci USA* **86**: 3838–3841

Dillner J, Lenner P, Lehtinen M, Eklund C, Heino P, Wiklund F, Hallmans G and Stendahl U (1994) A population-based seroepidemiological study of cervical cancer. *Cancer Res* **54**: 134–141

Dillner J, Wiklund F, Lenner P, Eklund C, Frederiksson SV, Schiller JT, Hibma M, Hallmans G and Stendahl U (1995) Antibodies against linear and conformational epitopes of human papillomavirus type 16 that independently associate with incident cervical cancer. *Int J Cancer* **60**: 377–382

Dillner L, Zellbi A, Åvall-Lundqvist E, Heino P, Eklund C, Pettersson CA, Forslund O, Hansson BG, Grandien M, Bistoletti P and Dillner J (1995) Association of serum antibodies against defined epitopes of human papillomavirus L1, E2, and E7 antigens and of HPV DNA with incident cervical cancer. *Cancer Detect Prev* **19**: 381–393

Fuchs PG, Girardi F and Pfister H (1989) Human papillomavirus 16 DNA in cervical cancers and in lymph nodes of cervical cancer patients: a diagnostic marker for early metastases? *Int J Cancer* **43**: 41–44

Galloway DA (1994) Papillomavirus Capsids: a new approach to identify serological markers of HPV infection. *J Natl Cancer Inst* **86**: 474–475

Gauthier J-M, Dillner J and Yaniv M (1991) Structural analysis of the human papillomavirus type 16-E2 transactivator with antipeptide antibodies reveals a high mobility region linking the transactivation and the DNA binding domains. *Nucl Acids Res* **19**: 7073–7079

Gissmann L and Muller M (1994) Serological immune response to HPV. In *Human Papillomavirus and Cervical Cancer*, Stern PL and Stanley MA. (eds), pp. 132–144. Oxford University Press: Oxford

King LA and Possee RD (1992) *The Baculovirus Expression System: A Laboratory Guide*. Chapman & Hall: London

Kochel HG, Sievert K, Monazahian M, Mittelstadt DA, Teichmann A and Thomssen R (1991) Antibodies to human papillomavirus type-16 in human sera as revealed by the use of prokaryotically expressed viral gene products. *Virology* **182**: 644–654

Lehtinen M, Leminen A, Paavonen J, Lehtovirta P, Hyoty H, Vesterinen E and Dillner J (1992*a*) Predominance of serum antibodies to synthetic peptide stemming from HPV 18 open reading frame E2 in cervical adenocarcinoma. *J Clin Pathol* **45**: 494–497

Lehtinen M, Leminen A, Kuoppala T, Tiikkainen M, Lehtinen T, Lehtovirta P, Punnonen R, Vesterinen E and Paavonen J (1992*b*) Pre- and post-treatment serum antibody responses to HPV 16 E2 and HSV 2 ICP8 proteins in women with cervical carcinoma. *J Med Virol* **37**: 180–186

Lenner P, Dillner J, Wiklund F, Hallmans G and Stendahl U (1995) Serum antibody responses against human papillomavirus in relation to tumor characteristics, response to treatment, and survival in carcinoma of the uterine cervix. *Cancer Immunol Immunother* **40**: 201–205

Mann VM, Loo De Lao S, Brenes M, Brinton LA, Rawls JA, Green M, Reeves WC and Rawls WE (1990) Occurrence of IgA and IgG antibodies to select peptides representing human papillomavirus type 16 among cervical cancer cases and controls. *Cancer Res* **50**: 7815–7819

Matsukura T, Koi S and Sugase M (1989) Both episomal and integrated forms of human papillomavirus type 16 are involved in invasive cervical cancers. *Virology* **172**: 63–72

Muller M, Viscidi RP, Sun Y, Guerrero E, Hill PM, Shah F, Bosch FX, Munoz N, Gissmann L and Shah KV (1992) Antibodies to HPV-16 E6 and E7 proteins as markers for HPV-16-associated invasive cervical cancer. *Virology* **187**: 508–514

Munoz N, Bosch FX, De Sanjose S, Tafur L, Izarzugaza I, Gili M, Viladiu P, Navarro C, Martos C, Ascunce N, Gonzalez LC, Kaldor JM, Guerrero E, Lorincz A, Santamaria M, Alonso De Ruiz P, Aristizabal N and Shah K (1992) The causal link between human papillomavirus and invasive cervical cancer: a population-based case-control study in Colombia and Spain. *Int J Cancer* **52**: 743–749

Nindl I, Benitez BL, Berumen J, Farmanara N, Fisher S, Gross G, Lopez CL, Muller M, Tommasino M, Vazquez-Curiel A and Gissmann L (1994) Antibodies against linear and conformational epitopes of the human papillomavirus (HPV) type 16 E6 and E7 oncoproteins in sera of cervical cancer patients. *Arch Virol* **137**: 341–353

Reeves WC, Rawls JA, Green M and Rawls WE (1990) Antibodies to human papillomavirus type 16 in patients with cervical neoplasia. *Lancet* **335**: 551–552

Sanders CM, Stern PL and Maitland NJ (1995) Characterization of human papillomavirus type 16 E2 protein and subdomains expressed in insect cells. *Virology* **211**: 418–433

Schiffman MH, Bauer HM, Hoover RN, Glass AG, Cadell DM, Rush BB, Scott DR, Sherman ME, Kurman RJ, Wacholder S, Stanton CK and Manos MM (1993) Epidemiologic evidence showing that human papillomavirus infection causes most cervical intraepithelial neoplasia. *J Natl Cancer Inst* **85**: 958–964

Seedorf K, Krammer G, Durst M, Suhai S and Rowekamp WG (1985) Human papillomavirus type 16 DNA sequence. *Virology* **145**: 181–185

Stacey SN, Bartholomew JS, Ghosh A, Stern PL, Mackett M and Arrand JR (1992) Expression of human papillomavirus type 16 E6 protein by recombinant baculovirus and use for detection of anti-E6 antibodies in human sera. *J Gen Virol* **73**: 2337–2345

Stacey SN, Eklund C, Jordan D, Smith NK, Stern PL, Dillner J and Arrand JR (1994) Scanning the structure and antigenicity of HPV-16 E6 and E7 oncoproteins using antipeptide antibodies. *Oncogene* **9**: 635–645

Stanley MA, Coleman N and Chambers M (1994) The host response to lesions induced by human papillomavirus. In *Vaccines Against Virally Induced Cancers: Ciba Foundation Symposium 187*, Chadwick DJ and Marsh J. (eds), pp. 21–44. John Wiley: Chichester

Turek LP (1994) The structure, function, and regulation of papillomaviral genes in infection and cervical cancer. *Adv Virus Res* **44**: 305–356

Viscidi RP, Sun Y, Tsuzaki B, Bosch FX, Munoz N and Shah KV (1993) Serologic response in human papillomavirus-associated invasive cervical cancer. *Int J Cancer* **55**: 780–784

Walboomers JMM, De Roda Husman A-M, Van Den Brule AJC, Snijders PJF and Meijer CJLM (1994) Detection of genital human papillomavirus infections: Critical review of methods and prevalence studies in relation to cervical cancer. In *Human Papillomavirus and Cervical Cancer*, Stern PL and Stanley MA. (eds), pp. 41–71. Oxford University Press: Oxford

© Cancer Research Campaign 1997